# Acute toxicities and cumulative dose to the brain of repeated sessions of stereotactic radiotherapy (SRT) for brain metastases: a retrospective study of 184 patients

**DOI:** 10.1186/s13014-022-02194-0

**Published:** 2023-01-10

**Authors:** L. Kuntz, C. Le Fèvre, D. Jarnet, A. Keller, P. Meyer, A. Thiery, H. Cebula, G. Noel, D. Antoni

**Affiliations:** 1 Radiation Therapy University Department, Institut de Cancérologie Strasbourg Europe (ICANS), 17 rue Albert Calmette, 67200 Strasbourg, France; 2 Medical Physics Unit, Institut de Cancérologie Strasbourg Europe (ICANS), 17 rue Albert Calmette, 67200 Strasbourg, France; 3 Medical Information Department, Institut de Cancérologie Strasbourg Europe (ICANS), 3 rue de la Porte de L’Hôpital, 67065 Strasbourg Cedex, France; 4 Department of Neurosurgery, University Hospitals of Strasbourg, 1 Avenue Molière, 67200 Strasbourg, France

**Keywords:** Salvage radiation, Stereotactic radiosurgery, Brain metastases, Side effect, Acute toxicity, Repeated radiosurgery

## Abstract

**Background:**

Stereotactic radiation therapy (SRT) is a focal treatment for brain metastases (BMs); thus, 20 to 40% of patients will require salvage treatment after an initial SRT session, either because of local or distant failure. SRT is not exempt from acute toxicity, and the acute toxicities of repeated SRT are not well known. The objective of this study was to analyze the acute toxicities of repeated courses of SRT and to determine whether repeated SRT could lead to cumulative brain doses equivalent to those of whole-brain radiotherapy (WBRT).

**Material and methods:**

Between 2010 and 2020, data from 184 patients treated for 915 BMs via two to six SRT sessions for local or distant BM recurrence without previous or intercurrent WBRT were retrospectively reviewed. Patients were seen via consultations during SRT, and the delivered dose, the use of corticosteroid therapy and neurological symptoms were recorded and rated according to the CTCAEv4. The dosimetric characteristics of 79% of BMs were collected, and summation plans of 76.6% of BMs were created.

**Results:**

36% of patients developed acute toxicity during at least one session. No grade three or four toxicity was registered, and grade one or two cephalalgy was the most frequently reported symptom. There was no significant difference in the occurrence of acute toxicity between consecutive SRT sessions. In the multivariate analysis, acute toxicity was associated with the use of corticosteroid therapy before irradiation (OR = 2.6; p = 0.01), BMV grade (high vs. low grade OR = 5.17; p = 0.02), and number of SRT sessions (3 SRT vs. 2 SRT: OR = 2.64; p = 0.01). The median volume equivalent to the WBRT dose (V_WBRT_) was 47.9 ml. In the multivariate analysis, the V_WBRT_ was significantly associated with the total GTV (p < 0.001) and number of BMs (p < 0.001). Even for patients treated for more than ten cumulated BMs, the median BED to the brain was very low compared to the dose delivered during WBRT.

**Conclusion:**

Repeated SRT for local or distant recurrent BM is well tolerated, without grade three or four toxicity, and does not cause more acute neurological toxicity with repeated SRT sessions. Moreover, even for patients treated for more than ten BMs, the V_WBRT_ is low.

## Introduction

Stereotactic radiation therapy (SRT) for brain metastases (BMs) has become a reference and seems to have mainly replaced whole-brain radiotherapy (WBRT) [1]. It has developed in recent years with increasingly broad indications [2]. Technical progress as well as advances in repositioning systems currently make it possible to treat BMs in complex situations, such as BMs in direct proximity to organs at risk, large BMs, multiple BM locations in one session, reirradiation for BM relapse or new metastasis in multiple sessions [3]. SRT provides the same overall survival benefit and local control while being less toxic at the cognitive and functional levels than WBRT [4–6]. SRT indications have evolved from one to four BMs to ten BMs if the cumulative volume is less than 30 ml [7–11]. With the increased life expectancy of patients with metastatic brain cancer due to advances in oncology and avoidance of WBRT, the rate of salvage SRT for distant brain relapse and reirradiation for local recurrence is increasing. Modalities of distant or local stereotactic reirradiation are poorly codified. The main issues are the number of BMs or the maximum volume allowed to be treated with SRT without compromising neurocognitive toxicity as with WBRT. Eventually, the tolerance of subsequent SRT is not as well documented as that of one SRT session or the first of multiple cumulative SRT sessions. This retrospective study analyzed 915 BMs from 184 patients who were not previously treated with WBRT and were treated with over two to six sessions of SRT. The objectives of this study were to analyze acute neurologic toxicity at each SRT session and to determine whether repeated SRT sessions could lead to cumulative brain doses equivalent to those of WBRT.

## Materials and methods

### Brain metastasis and dosimetry plans

Between January 2010 and June 2020, 915 BMs were treated with SRT among 184 patients. Patients have between two to six sessions of SRT without previous or intercurrent WBRT. Planning Target Volume (PTV) was obtained by adding a circumferential margin of two millimeters around the Gross Tumor Volume (GTV). Monofractionated treatment delivering 20 Gy at the isocenter prescribed on the 70% isodose line was used for 26% of BMs. Hypofractionated treatment delivering three fractions of 11 Gy at the isocenter prescribed on the 70% isodose line every two days was used for 72% of BMs. Other hypofractionated schedules, such as 10 × 3 Gy or 5 × 6.25 Gy, were used for 2% of BMs. Treatment plans were calculated on the iPlan RT Plan (Brainlab) or Eclipse® System (Varian Medical Systems®, Palo Alto, CA, USA). Patients for whom one or more treatment plans were no longer available in the archives were excluded from the dosimetric analysis. The dosimetry plans of 722 BMs (79%) could be collected, representing 141 (76.6%) patients. All dosimetric scans and contoured volumes were transferred to an Eclipse® System (Varian Medical Systems®, Palo Alto, CA, USA). For each BM, the GTV and PTV were collected in ml. For each patient, a summation plan, including all the treatment plans, was created by merging the dosimetric CT findings from each session. The dosimetric CT finding from the first SRT session was used as a reference. From the 76.6% of available summation dosimetric plans, the mean brain doses; mean healthy brain doses; V_12Gy_, V_14Gy_, V_21Gy_, and V_23.1 Gy_ to the brain; and V_WBRT_ were collected. The V_WBRT_ was defined as the brain-V_19.2 Gy_ in the trifractionated scheme and the brain-V_12Gy_ in the monofractionated scheme and was equivalent to a BED in WBRT.

### Medical consultations during radiotherapy

Patients were seen via a consultation with the referring radiation oncologist after a multidisciplinary discussion that established the radiotherapy plan. Information on the clinical history, oncological treatments, and use of corticosteroid therapy, as well as the different neurological symptoms of the patients, was collected. During SRT, patients were seen weekly by a radiation oncologist. At the request of the patient or of the paramedical team, the patient could be seen by the physician in addition to the mandatory consultations [12]. At each consultation during radiotherapy treatment, the delivered dose, the use of corticosteroid therapy and neurological symptoms (headache, nausea, epilepsy, confusion, and sensorimotor disorders) were recorded and rated according to the CTCAE v4 [13]. Follow-up MRI were performed every three to six months after SRT to measure therapeutic efficiency, diagnose local recurrence (LR), cerebral recurrence or radionecrosis. A new contrast enhancement outside the previously treated BM was categorized as a cerebral recurrence [14]. A contrast enhancement inside the previously treated BM suggested a LR. Confirmation of LR was made by surgery or complementary examination, such as 18-FDG-PET-CT, F-DOPA-PET-CT, or a new MRI performed in a shorter interval [15–18].

### Statistical analysis

Quantitative variables are described using standard position and dispersion statistics, namely, the mean, median, variance, minimum, maximum and quantiles. Qualitative data are described as the numbers and proportions of each modality. Cumulative proportions were also calculated for variables with more than two modalities. The Gaussian distribution of the quantitative variables was assessed using the Shapiro–Wilk test. If the conditions were met, the relationship between two quantitative variables was assessed using Pearson's linear correlation test. Otherwise, a Spearman correlation test was used. For the comparison of a quantitative variable between several subgroups, analysis of variance was used. For the comparison of a quantitative variable between several subgroups, analysis of variance or the Kruskal–Wallis test was used (again, according to the assumptions of the use of each of these tests). Finally, for the comparison between several qualitative variables, the parametric chi2 test was used if the conditions of the application allowed it. If this was not the case, Fisher’s exact test was used. The alpha risk was set at 5% for all analyses. All analyses were performed using R software version 3.1, R Development Core Team (2008, R Foundation for Statistical Computing, Vienna, Austria) and GMRC Shiny Stat (2017).

## Results

### Patient and BM characteristics

A total of 184 patients were enrolled: 103 (56%) were followed for lung cancer, 24 (13%) for breast cancer, 24 (13%) for melanoma, 16 (8.7%) for digestive cancer, eight (4.3%) for kidney cancer, and nine (4.9%) for other cancers. Sixty-nine (37.5%) patients were diagnosed with initial brain metastases. At SRT1 (SRT session n°1), the median patient age was 61 (24–88) years, the median WHO grade was one (0–3), the median KPS score was 90% (40–100%), and the median DS-GPA was 2.5 (0–4). BMV grade was classified as low, intermediate, or high for 103 (56%), 69 (37.5%) and twelve (6.5%) patients, respectively. Immunotherapy and targeted therapy were given to 25 (13.6%) and 43 (23.4%) patients during at least one SRT session, respectively. Patient and primary tumor characteristics are shown in Table 1.

**Table 1:** Patient characteristics (n = 184)

*Characteristic*			
Sex	Male	91	49.5%
	Female	93	50.5%
Control of the primary tumor site	Yes	109	59.2%
	No	75	40.8%
Primary cancer	Lung	103	56%
	Adenocarcinoma	*69*	*67%*
	Epidermoid	*19*	*18.4%*
	Small cell	*4*	*3.9%*
	Undifferentiated	*4*	*3.9%*
	Other	*7*	*6.8%*
	Breast	24	13%
	Luminal A	*5*	*20.8%*
	Luminal B	*5*	*20.8%*
	Her2 +	*11*	*45.8%*
	Triple negative	*3*	*12.5%*
	Melanoma	24	13%
	Kidney	8	4.35%
	Digestive	16	8.7%
	Other	9	4.95%
Patient characteristics at SRT1	Median age (range)	61	24–88
	Median WHO grade (range)	1	0–3
	Median KPS score in % (range)	90	40–100
	Median DS-GPA (range)	2.5	0–4
	Median RPA (range)	2	1–3
	Extracerebral progression	117	64%
	Systemic treatment	129	70%
	Median number of BMs (range)	1.5	1–10
	Median volume of BMs (in mL) (range)	0.8	0.1–48.6
	Tumor bed radiosurgery	70	38%
Patient’s worst/all characteristics	Worst DS-GPA	2	0–4
	Worst RPA	2	1–3
	Immunotherapy all time	25	14%
	Targeted therapy all time	43	23%
	BMV grade		
	Low	103	56%
	Intermediate	69	37.5%
	High	12	6.5%
	Salvage WBRT	34	18%
	Local recurrence	57	31%
	Radionecrosis	42	23%

Italic values represent a subgroup of the set cited in the row above

One hundred and twenty-three (66.8%) patients received two sessions of SRT, 39 (21.2%) received three sessions, and 22 (12%) received four or more sessions, for a total of 461 administered treatments. Each patient had a mean of two BMs treated per SRT session (range: 1–6; 95% CI 1.88–2.12), for a total average of five BMs treated during all sessions (range: 2–19; 95% CI 4.52–5.44). Nine hundred and fifteen BMs were treated between 07/09/2010 and 06/15/2020. Eight hundred and nineteen BMs (89.5%) were irradiated in place, and 96 tumor bed (10%) were irradiated after surgery. Fifty-one (5.6%) BMs were reirradiated after a confirmation of local recurrence. Six hundred and seventy-eight (74.1%) BMs were supratentorial, and 321 (24.2%) were infratentorial (the locations of 16 (1.7%) were unknown). Two hundred and thirty-five (25.7%) BMs received monofractionated treatment delivering 20 Gy in one fraction at the isocenter, with the 70% isodose (14 Gy) encompassing the PTV, and 659 (72%) BMs received hypofractionated treatment delivering 33 Gy in three fractions of 11 Gy at the isocenter, with the 70% isodose (23.1 Gy) encompassing the PTV. Twenty-one (2.3%) BMs received hypofractionated treatment, such as 10 × 3 Gy or 5 × 6.25 Gy. Characteristics of the BMs are shown in Table 2.

**Table 2:** Brain metastases characteristics (n = 915)

Localization	Supratentorial	678	74%
	Frontal	*279*	*30%*
	Parietal	*147*	*16%*
	Temporal	*93*	*10%*
	Occipital	*107*	*12%*
	Central	*52*	*6%*
	Infratentorial	213	23%
	Cerebellar	*211*	*23%*
	Cerebellopontine angle	*2*	*0%*
	Brainstem	8	1%
	Unknown	16	2%
Side	Right	459	50%
	Left	417	46%
	Median	24	3%
	Unknown	15	2%
Neurosurgery	Yes	96	10%
	Tumor residue	*15*	*16%*
	No	819	90%
Prescribed doses	20 Gy in 1 fraction	235	26%
	33 Gy in 3 fractions	659	72%
	Other	21	2%

*BM* brain metastasis, *BMV* brain metastases velocity, *DS-GPA* Diagnosis-Specific Graded Prognostic Assessment, *KPS* Karnofsky Performance Score, *RPA* recursive partitioning analysis, *SRT1* first session of stereotactic radiotherapy, *WBRT* whole-brain radiotherapy

Italic values represent a subgroup of the set cited in the row above

### Volumetric characteristics of BMs

The median GTV per BM was 0.4 ml (95% CI mean 2.47–3.48). The median GTVs treated per BM at SRT1, SRT2, SRT3 and SRT4 and thereafter were 0.4, 0.4, 0.25 and 0.35 ml, respectively (p = 0.51). The median GTVs per session (i.e., the sum of GTVs treated during an SRT session) were 6.2 ml (95% CI mean 7.01–9.9), 0.9 ml (95% CI mean 3.3–6.8), 0.6 ml (95% CI mean 1.3–4.8) and 1 ml (95% CI mean 0.1–10.2) at SRT1, SRT2, SRT3, and SRT4 and thereafter, respectively. The GTV at SRT1 was significantly higher than those of the subsequent SRT sessions (p < 0.001), and there was no difference between the GTVs at SRT2 and at SRT3 or between the GTVs at SRT3 and at SRT4 and thereafter, with p = 0.34 and 0.62, respectively. The median GTV_sum_ (i.e., the sum of the GTVs of all BMs in all SRT sessions) was 10.7 ml (95% CI mean 12.78–17.73). The median PTV_sum_ (i.e., the sum of the PTVs of all BMs in all SRT sessions) was 22.8 ml (95% CI mean 24.14–31.84). The PTV_sum_ represents a median of 1.55% of the brain (95% CI mean 1.75–2.33). There was a statistically significant difference between the PTV at SRT1 and the PTV following SRT (p < 0.001).

### Neurologic symptoms

One hundred and three (56%), 25 (41%) and ten (45.5%) patients presented with neurologic symptoms preirradiation at SRT1, SRT2, SRT3 and SRT4 and thereafter, respectively (p = 1). Sixty-seven (36.4%), 74 (40.2%), 21 (34.4%) and eight (36.4%) patients developed no neurologic symptoms up to the last ongoing consultation for SRT1, SRT2, SRT3 and SRT4 and thereafter. There was a statistically significant difference in neurologic symptom prevalence in the before-after analysis at SRT1 and SRT2 (p < 0.01 and p < 0.01, respectively). There was no statistically significant difference in neurologic symptom prevalence in the before-after analysis at SRT3 and SRT4 and thereafter (p = 0.38 and p = 0.68, respectively). Sixty-three (34.2%), 67 (36.4%), 17 (27.9%) and six (27.3%) patients received corticosteroid therapy before SRT1, SRT2, SRT3 and SRT4 and thereafter, respectively. A decrease, no change or increase in neurological symptoms was observed for 48 (26.1%), 123 (66.8%) and 13 (7.1%) patients after SRT1, 40 (21.7%), 133 (72.3%), and eleven (6.0%) after SRT2, eight (13.1%), 49 (80.3%), and four (6.6%) after SRT3, and four (18.2%), 16 (72.7%), and two (9.1%) after SRT4.

One hundred and seventy (92.4%), 156 (84.8%), 53 (86.9%), and 17 (77.3%) patients showed no signs of confusion just before SRT1, SRT2, SRT3 and SRT4 and thereafter, respectively. One hundred and seventy-nine (97.3%), 168 (91.3%), 55 (90.2%), and 19 (86.4%) patients showed no signs of confusion after SRT1, SRT2, SRT3 and SRT4 and thereafter, respectively. There was a statistically significant difference in confusion grade before SRT1 and before SRT2 (p = 0.03) and before-after SRT1 (p = 0.02) and SRT2 (p = 0.02). One hundred and sixty-two (88%), 157 (85.3%), 56 (91.8%), and 20 (90.9%) patients showed no signs of epilepsy before SRT1, SRT2, SRT3 and SRT4 and thereafter, respectively [13]. One hundred and seventy-four (94.6%), 169 (91.8%), 58 (95.1%), and 21 (95.5%) patients showed no signs of epilepsy after SRT1, SRT2, SRT3 and SRT4 and thereafter, respectively. A statistically significant difference was found in the before-after analysis of SRT1 (p = 0.02) and SRT2 (p = 0.05). One hundred and eighteen (64.1%), 114 (62%), 44 (72.1%), and 17 (77.3%) patients showed no signs of sensorimotor disorders before SRT1, SRT2, SRT3 and SRT4 and thereafter, respectively. One hundred and forty (76.1%), 133 (72.3%), 46 (75.4%), and 18 (81.8%) patients showed no signs of sensorimotor disorders after SRT1, SRT2, SRT3 and SRT4 and thereafter, respectively. A statistically significant difference was found in the before-after analysis of SRT1 (p < 0.01) and SRT2 (p < 0.01). Table 3 shows acute neurologic symptoms according to the CTCAE (v4) at each SRT session, and Table 4 shows the p value of the before-before and before-after symptom comparisons between each SRT session.

**Table 3:** Acute symptoms according to the CTCAE (v4) at each SRT session

	SRT1 (n = 184)		SRT2 (n = 184)		SRT3 (n = 61)		SRT ≥ 4 (n = 31)	
	Number of patients	Percentage (%)	Number of patients	Percentage (%)	Number of patient	Percentage (%)	Number of patients	Percentage (%)
*Symptoms pre-SRT*								
Yes	102	55	103	56	25	41	13	42
No	82	45	81	44	36	59	18	58
*Symptoms post-SRT*								
Yes	67	36	74	40	21	34	13	42
No	117	64	110	60	40	66	18	58
*Corticotherapy pre-SRT*								
Yes	63	34	67	36	17	28	11	35
No	121	66	117	64	44	72	20	65
*Cephalalgy pre-SRT*								
Grade 0	136	74	132	72	47	77	26	84
Grade 1	36	20	41	22	11	18	4	13
Grade 2	12	7	11	6	2	3	1	3
Grade 3	0	0	0	0	1	2	0	0
*Cephalalgy post-SRT*								
Grade 0	136	74	132	72	46	75	25	81
Grade 1	36	20	43	23	10	16	5	16
Grade 2	12	7	8	4	4	7	1	3
Grade 3	0	0	1	1	1	2	0	0
*Confusion pre-SRT*								
Grade 0	170	92	156	85	53	87	25	81
Grade 1	11	6	21	11	6	10	5	16
Grade 2	3	2	7	4	2	3	1	3
*Confusion post-SRT*								
Grade 0	179	97	168	91	55	90	27	87
Grade 1	5	3	11	6	3	5	4	13
Grade 2	0	0	5	3	3	5	0	0
*Nausea pre-SRT*								
Grade 0	175	95	168	91	59	97	29	94
Grade 1	5	3	14	8	1	2	1	3
Grade 2	4	2	2	1	1	2	1	3
*Nausea post-SRT*								
Grade 0	171	93	173	94	59	97	28	90
Grade 1	9	5	10	5	1	2	2	6
Grade 2	4	2	1	1	1	2	1	3
*Epilepsy pre-SRT*								
Grade 0	162	88	157	85	56	92	28	90
Grade 1	15	8	21	11	3	5	3	10
Grade 2	7	4	6	3	2	3	0	0
*Epilepsy post-SRT*								
Grade 0	174	95	169	92	58	95	30	97
Grade 1	6	3	10	5	1	2	1	3
Grade 2	4	2	5	3	2	3	0	0
*Sensorimotor disorder pre-SRT*								
Grade 0	118	64	114	62	44	72	23	74
Grade 1	29	16	38	21	5	8	4	13
Grade 2	37	20	32	17	11	18	4	13
Grade 3	0	0	0	0	1	2	0	0
*Sensorimotor disorder post-SRT*								
Grade 0	140	76	133	72	46	75	25	81
Grade 1	21	11	27	15	7	11	4	13
Grade 2	23	13	23	13	7	11	2	6
Grade 3	0	0	1	1	1	2	0	0

**Table 4:** Before–before and before–after symptom comparisons (McNemar test)

	Before-before SRT1/SRT2	Before-before SRT2/SRT3	Before-after SRT1	Before-after SRT2	Before-after SRT3	Before-after SRT 4/5/6
Any symptom	1	1	** < 0.01**	** < 0.01**	0.38	0.68
Cephalalgy	0.39	NA	0.68	1	1	1
Confusion	**0.03**	0.54	**0.02**	**0.02**	0.61	0.61
Nausea	0.11	NA	0.65	0.22	1	1
Epilepsy	0.7	NA	**0.02**	**0.05**	0.61	1
Sensorimotor disorder	0.49	NA	** < 0.01**	** < 0.01**	0.72	1

*SRT* stereotactic radiotherapy, *NA* not assessable

Bold values indicate significant values

### Factors associated with neurologic symptoms

Eighty-one (44%) patients had no immediate neurological symptoms during any treatment session. In the univariate analysis, only the worst RPA for all SRT sessions was associated with the presence of neurologic symptoms (p = 0.02). Neither the primary cancer (p = 0.24), patient history (with p = 0.54, 0.13 and 0.8 for a history of alcohol, tobacco and diabetes, respectively), the total number of BMs (p = 0.38), the GTV_sum_ of BMs (p = 0.61), the number of SRT sessions (p = 0.47), immunotherapy (p = 1), targeted therapy (p = 0.71), or surgery (p = 0.77) was associated with neurologic symptoms. The intake of corticosteroid therapy was associated with post-SRT symptom development (p = 0.02).

### Acute toxicity

#### Acute toxicity at each SRT session

No patient had grade 4 neurologic symptoms pre- or post-SRT during any SRT session. One patient developed grade three cephalalgy pre- and post-SRT3, one patient developed a grade three sensorimotor disorder pre- and post-SRT3, and no patient developed any new grade three acute toxicity. New or worsening neurologic symptoms developed in 33 (17.9%), 40 (21.7%), ten (16.4%), and five (16.1%) patients after SRT1, SRT2, SRT3 and SRT4 and thereafter, respectively. Among them, grade one and two cephalalgy was the most frequently reported symptom in 17 (51.5%), 21 (52.5%), and six (60%) patients at SRT1, SRT2, and SRT3, respectively. Other symptoms were sensorimotor disorder, followed by nausea, epilepsy, and confusion, with respective incidences of 27%, 18%, 16% and 8%. There was no significant difference in the occurrence of acute toxicity between each consecutive SRT session, with p = 0.37, p = 0.48 and p = 0.62 between SRT1-SRT2, SRT2-SRT3 and SRT3-SRT4 and thereafter, respectively. At SRT2, patients with a poor performance status (advanced WHO grade, low KPS score, low DS-GPA and high RPA) tended to have more acute toxicities than their counterparts. At SRT3, the association between toxicity and a poor performance status was statistically significant, with p = 0.01, p < 0.01, p = 0.04 and p = 0.05 for WHO grade, KPS score, DS-GPA and RPA, respectively. In the univariate analysis, the number of BMs irradiated was not significantly associated with the occurrence of toxicity at any treatment session. However, there was a statistical trend in the association between BM volume at each session and the occurrence of toxicity at SRT1, SRT2, SRT3 and SRT4 and thereafter (p = 0.07, p = 0.3, p = 0.05 and p = 0.11, respectively). There were no significant associations between acute toxicity at each SRT session and immunotherapy or targeted therapy. Patients who experienced acute toxicity at SRT1 were statistically more likely to also experience acute toxicity at SRT2 (p < 0.01), and this association was not found for subsequent sessions at SRT3 and SRT4 (p = 1 and p = 1, respectively). In the multivariate analysis, there was no statistical association between acute toxicity at each SRT session and clinical or dosimetric characteristics. Table 5 shows uni- and multivariate associations between acute toxicity during each SRT session and patient and dosimetric characteristics.

**Table 5:** Association between acute toxicity at each SRT session and clinical and dosimetric characteristics

	SRT1 (n = 184)		SRT2 (n = 184)		SRT3 (n = 61)	
	U	M	U	M	U	M
*Patient characteristics*						
Age	0.44	0.38	**0.01**	0.87	0.43	1
WHO grade	0.99	0.76	0.07	0.73	**0.01**	1
KPS score	0.11	0.31	0.15	0.85	** < 0.01**	1
DS-GPA	0.99		0.34		**0.04**	
RPA	0.06		0.62		**0.05**	
Symptoms pre-SRT	0.3	0.46	**0.04**	0.45	0.29	1
Corticosteroid use pre-SRT	0.78	0.85	** < 0.01**	0.07	0.12	1
Immunotherapy	0.27	0.32	0.36	0.37	0.29	1
Targeted therapy	0.78	0.74	0.62	0.06	0.67	1
*BM characteristics*						
Number of BMs	0.18	0.89	0.09	0.1	0.66	1
Surgery	0.35		1		0.6	
GTV	0.07	0.13	0.30	0.67	**0.05**	1
Local recurrence	/		0.96		0.17	

*BM* brain metastasis, *DS-GPA* Diagnosis-Specific Graded Prognostic Assessment, *GTV* gross tumor volume, *KPS* Karnofsky Performance Score, *M* multivariate analysis, *RPA* recursive partitioning analysis, *SRT* stereotactic radiotherapy, *U* univariate analysis

Bold values indicate significant values

#### Cumulative acute toxicity

Cumulative acute toxicity was defined as the presence or absence of any neurologic acute toxicity during at least one session of repeated SRT. Sixty-seven (36.4%) patients developed cumulative acute toxicity. Cumulative acute toxicity was significantly associated with the number of SRT sessions (p = 0.01) and tended to be associated with the total number of BMs (p = 0.08). Cumulative acute toxicity was not associated with patient history, the GTV_sum_, or the isodose of summation dosimetry. In the multivariate analysis, corticosteroid therapy was the only factor that was significantly associated with cumulative acute toxicity (p = 0.03). Immunotherapy and DS-GPA tended to be associated with cumulative acute toxicity (p = 0.06 and p = 0.10, respectively). Table 6 shows the association between cumulative acute toxicity during all SRT sessions and patient and dosimetric characteristics.

**Table 6:** Association between cumulative acute toxicity and clinical and dosimetric characteristics

		p value univariate	p value multivariate
Patient’s history and tumor characteristics	Sex	0.51	0.14
	Hypertension	0.68	0.25
	Kidney disease	0.54	
	Cholesterol	1	
	Tobacco	0.52	0.22
	Alcohol	0.12	0.21
	Diabetes	0.25	0.99
	Primary tumor	0.24	
Patient’s worst/all characteristics	Worst DS-GPA	0.49	0.10
	Worst RPA	0.67	0.20
	Corticosteroid therapy before SRT	** < 0.001**	**0.03**
	Immunotherapy all time	0.2	0.06
	Targeted therapy all time	0.63	0.53
	BMV grade	**0.04**	0.13
	Surgery	0.30	0.39
	Local reirradiation	0.45	0.28
	Symptoms pre-SRT at any session	** < 0.001**	0.21
Dosimetric characteristics	Number of SRT sessions	**0.01**	0.35
	Number of SRT sessions > 3	**0.04**	
	Total number of BMs	0.08	0.67
	BMs > 5	0.28	
	BMs > 10	0.59	
	Total GTV	0.61	0.15
	V_12Gy_	0.52	
	V_14Gy_	0.58	
	V_21Gy_	0.64	
	V_23Gy_	0.6	
	V_40Gy_	0.51	

*BM* brain metastasis, *BMV* brain metastasis velocity, DS-GPA Diagnosis-Specific Graded Prognostic Assessment, *GTV* gross tumor volume, *KPS* Karnofsky Performance Score, *RPA* Recursive Partitioning Analysis, *SRT* stereotactic radiotherapy

Bold values indicate significant values

### Cumulative dose to the brain

The absolute cumulative median dose delivered to the brain (D_mean brain_) was 4.28 Gy (95% CI mean 4.61–5.57), corresponding to 5.31 Gy _BED_ (95% CI mean: 5.91–7.37) with an α/β ratio of 3. The absolute median dose to the healthy brain (D_mean healthy brain_) was 3.98 Gy (95% CI mean 4.33–5.22), corresponding to 4.92 Gy _BED_ (95% CI mean 5.47–6.81). Fifty-three (28.8%) patients received at least one session delivered in multi-isocentric mode. In the univariate analysis, the BED_mean brain_ and BED_mean healthy brain_ were significantly associated with the multi-isocentric radiotherapy technique (p < 0.001), total number of BMs (p < 0.001), total GTV and PTV (p < 0.001) and volume of the largest BM (p < 0.001). The BED_mean brain_ and BED_mean healthy brain_ showed a trend toward a significant association with at least one resected BM (p = 0.07 and p = 0.1, respectively) and the number of SRT sessions (p = 0.14 and p = 0.13, respectively). In the multivariate analysis, the BED_mean brain_ and BED_mean healthy brain_ were significantly associated with the GTV_sum_ (p < 0.001), total number of BMs (p < 0.001) and total number of SRT sessions (p < 0.001). If a WBRT scheme of 30 Gy in ten fractions was prescribed, the normal brain BED (α/β = 3 Gy) would be 60 Gy. The V_19.2 Gy_ in the trifractionated scheme and the V_12Gy_ in the monofractionated scheme were equivalent to a BED of WBRT (V_WBRT_). The median V_WBRT_ was 47.9 ml (3.4% of brain volume). In the univariate analysis, the V_WBRT_ was statistically dependent on the number of SRT sessions (p = 0.047) and GTV_sum_ (Pearson correlation: r = 0.7) but not on the number of BMs (p = 0.23) or on the multi-isocentric RT technique (p = 0.11). In the multivariate analysis, the V_WBRT_ was significantly associated with the GTV_sum_ (p < 0.001) and number of BMs (p < 0.001) but not with the number of SRT sessions (p = 0.68) or the multi-isocentric RT technique (p = 0.14). For patients who were treated for more than ten BMs, although the cumulative V_12Gy_, V_14Gy_, V_21Gy_, and V_23.1 Gy_ were above the usually accepted constraints for a single session of SRT, the mean V_WBRT_ was only 101.4 ml (6.8% of the brain) [19–23].

## Discussion

Our retrospective study addressed repeated SRT sessions for BMs in patients with local or distant brain recurrence without previous WBRT. The purpose was primarily to analyze neurological symptoms and acute toxicity during each SRT session and the cumulative risk of acute toxicity during any SRT session and secondly to analyze the cumulative dose to the brain.

Few studies have examined acute toxicity during SRT, and most of these studies were conducted on a single SRT session or even irradiation to a single BM [24–26]. Studies of repeated SRT sessions have paid little attention to acute toxicity during radiotherapy [27–34].

This is the first study to address the potential impact of repeated irradiation to multiple BMs. Moreover, no study has investigated the dose to the brain and to the healthy brain in cases of repeated SRT [35].

First, this study included many patients and a large number of BMs. The neurological symptoms experienced by patients were recorded before and after irradiation. The monocentricity of this study appears to be an advantage, since it allowed us to collect the same symptoms at each session, have very few missing data and use a homogeneous prescription dose for most patients. We highlight that all the neurological symptoms collected, except for confusion, experienced by the patients before irradiation were stable over time. This observation is comparable to those reported in the study of Kuntz et al. based on the same population, which showed that WHO grade, DS-GPA and RPA were stable between each session of SRT (article in submission).

Then, only grade one and two confusion were increased at SRT2 compared to SRT1. However, this increase is likely multifactorial. This higher confusion rate can be explained by age, progressive brain disease, corticosteroid therapy, and leukoencephalopathy [36, 37]. Indeed, confusion before SRT1 tends to be more frequent for patients older than 65 years. The occurrence of ≥ grade one confusion was 5% for patients aged under 65 years and 13.6% for patients aged 65 years and over (p = 0.17). Chemotherapy-induced cognitive impairment (chemobrain) could also explain this increased confusion from SRT2, although it was not representative in our series [38]. A prospective study would certainly have allowed us to better distinguish the toxicity related to SRT itself from that of systemic treatments and to better take into account the effect of corticosteroid therapy. But despite the fact that our study is retrospective, we have few missing clinical and dosimetric data. The monocentricity of our study may appear as a weakness, but it allows us a certain homogeneity in the prescription of the radiotherapy dose, the clinical follow-up during radiotherapy and the radiological follow-up after the end of the radiotherapy. Thirty-seven percent of patients were treated with immunotherapy or targeted therapy concomitantly with radiotherapy under stereotactic conditions during almost one SRT session, the specific toxicity due to systemic treatments could not be assessed. However, in temporal analyses of acute toxicity during SRT (Tables 3 and 4, before/before and before/after analysis) each patient was compared to himself, thus negating the potential effect of systemic treatments in the patients concerned. Furthermore, the analyses of acute toxicity at each session and of cumulative toxicity over time did not reveal any link between systemic treatments and the neurological symptoms studied, either in univariate or in multivariate analysis.

Furthermore, by taking an interest in the neurological status of patients before and after stereotaxis, it is realized that symptoms such as epilepsy, sensorimotor disorders and confusion are less important after treatment. This finding is most likely related to the effect of corticosteroid therapy, which was systematically prescribed during SRT in our center.

Moreover, no patient developed grade three or four acute toxicity, and the incidence of acute toxicity was stable over the course of the SRT sessions. According to the European Association of Neuro-Oncology, multiple courses of SRT for new BMs after an initial course of SRT with avoidance of WBRT could provide a low risk of toxicity [39]. Our acute toxicity results are similar to those reported in the study by Jimenez et al. [24] Indeed, they studied the acute toxicity of SRT among 156 patients treated for a single BM. Twenty-four percent of patients experienced at least one adverse symptom potentially associated with SRT, and the most common symptoms were fatigue and headache. In the final report of RTOG protocol 90–05, toxicity was significantly associated with the maximum GTV diameter, and larger tumors had a greater risk of unacceptable neurotoxicity than smaller tumors [40]. However, in this trial, the patients were reirradiated. The data did not show a correlation between local reirradiation and acute toxicity. Among the 184 patients, 58% received corticosteroid therapy before at least one SRT session because of symptomatic BM. Corticosteroid therapy limits the edema induced by BM [41, 42] and the early edema induced by SRT [43], but it can also increase confusion, especially in elderly patients [44]. Indeed, symptomatic patients tend to be older, have a poorer performance status, and tend to have more numerous and larger BMs.

Finally, we studied the cumulative dose to the brain. The objective was to determine whether repeated SRT sessions could lead to cumulative brain doses equivalent to those of WBRT. SRT is known to cause less cognitive deterioration than WBRT, even for patients treated for up to ten BMs [45–50], but no study has examined the neurocognitive impact of repeated SRT sessions. Hatiboglu et al. simulated different treatment plans by varying tumor volumes, tumor numbers and prescribed doses for patients treated for BMs by SRT [51]. The GTV_sum_ was a good predictor of the mean whole brain dose. We found the same results in the case of repeated SRT sessions, as the BED_mean brain_ and BED_mean healthy brain_ and V_WBRT_ were significantly associated with the GTVs_um_ and total number of BMs.

However, progressive BM per se can impact cognitive function. A prospective trial with dedicated neurocognitive tests would be desirable to better evaluate the impact of repeated SRT sessions on cognition and possibly compare repeated SRT to WBRT with hippocampal preservation.

## Conclusion

In this retrospective study on 184 patients treated for 915 BMs by repeated postoperative stereotactic radiosurgery or stereotactic radiotherapy for locally or cerebral recurrent BMs, 36% of patients developed acute toxicity during at least one session of repeated SRT. No grade three or four toxicity was reported, and grade one or two cephalalgy was the most frequently reported toxicity. There was no significant difference in the occurrence of acute toxicity between consecutive SRT sessions. The V_WBRT_ was significantly associated with the GTV_sum_ (p < 0.001) and number of BMs (p < 0.001). Even in patients who were treated for more than ten BMs, the V_WBRT_ remained low.

## Data Availability

Not applicable.

## Abbreviations

BM: Brain metastasis
BED: Biological equivalent dose
BMV: Brain metastases velocity
DS-GPA: Diagnosis-Specific Graded Prognostic Assessment
GTV: Gross tumor volume
KPS: Karnofsky Performance Score
LR: Local recurrence
OS: Overall survival
PTV: Planning target volume
RPA: Recursive partitioning analysis
SRT: Stereotactic radiotherapy
SRT1: First session of stereotactic radiotherapy
SRT2: Second session of stereotactic radiotherapy
SRT3: Third session of stereotactic radiotherapy
SRT4: Fourth session of stereotactic radiotherapy
WBRT: Whole-brain radiotherapy
WHO: WHO Performans status
